# 

**Published:** 1849-06

**Authors:** 


### CONTENTS

OF NO. VI.

---

### ORIGINAL COMMUNICATIONS.

ESSAYS AND CASES.

Art. I.—Remarks on some of the more Prevalent Diseases of Ohio in 1848. By John Dawson, M.D., of Jamestown, Ohio .......... 461
Art. II.—Lectures on Typhoid Fever, delivered in the Hotel Dieu Hospital of Lyons. By M. Brachet. Translated by D. W. Yandell, M.D., of Louisville, Ky..... 487
Art. III.—An Anomalous Case. By William H. Miller, M.D., of Louisville, Ky.......... 495
Art. IV.—An Account of some Rare Cases of Disease. By W. L. Sutton, M.D., of Georgetown, Ky.......... 497
Art. V.—A Notice of Cholera as it appeared in Louisville in May, 1849. By Theodore S. Bell, M.D.......... 510

SELECTIONS FROM AMERICAN AND FOREIGN JOURNALS.

Report of the Committee on Hygiene of Paris, in view of the approach of Cholera .......... 521
Oil of Naphtha in Diarrhea.......... 524
External use of Chloroform in Lumbago.......... 525
Puncture of the Bladder.......... 530
Calomel in the Treatment of Cholera.......... 533
Cod-liver Oil.......... 535
Fracture of the Penis .......... 536
Kreosote in Erysipelas.......... 536
Quantity of Carbonic Acid exhaled by Man in Health and Disease.. 537
Vigo's Plaster in Small-Pox .......... 539
Galvanism in Paralysis of the Bladder.......... 539

Nocturnal Visits........................................540
On the External Use of Iodine in Croup...................540
Chloroform in Mania-a-potu...............................540

ORIGINAL INTELLIGENCE.

The American Medical Association.........................541
National Convention for Revising the Pharmacopeia of the U. S..542
Congress Report on the Discovery of the Letheon..........543
Castor Oil and Spirits of Turpentine in Typhiod Fever....544
Chloroform in Labor......................................544
Progress of Cholera......................................544
Treatment of Cholera.....................................547
Professor Silliman.......................................548
Number of Chairs in Medical Schools......................548
Professor of Surgery in Harvard University...............549
Meig's Obstetrics........................................549
Correction...............................................549

